# Management and attitudes about IPF (Idiopathic Pulmonary Fibrosis) among physicians from Latin America

**DOI:** 10.1186/s12890-017-0569-1

**Published:** 2018-01-10

**Authors:** Iván Cherrez-Ojeda, Vincent Cottin, Juan Carlos Calderón, César Delgado, Erick Calero, Daniel Simanca-Racines, Silvia Quadrelli, Annia Cherrez

**Affiliations:** 1 Universidad de Especialidades Espíritu Santo, Samborondón, Ecuador; 2 Respiralab, Respiralab Research Group, Guayaquil, Ecuador; 3 Louis Pradel Hospital, Reference center for rare pulmonary diseases, Auvernia-Ródano-Alpes, Lyon, France; 4 Hospital Metropolitano, Quito, Ecuador; 5 Centro de Investigación en Salud Pública y Epidemiología Clínica. Facultad de Ciencias de la Salud Eugenio Espejo. Universidad Tecnológica Equinoccial., Quito, Ecuador; 6 Hospital Británico, Buenos Aires, Argentina; 7 Sanatorio Güemes, Buenos Aires, Argentina; 8 University of Heidelberg, Heidelberg, Germany

**Keywords:** Idiopathic pulmonary fibrosis, Health knowledge, Attitudes, Practice, Surveys and questionnaires, Latin America, Physicians

## Abstract

**Background:**

The aim of our study was to assess current practice patterns and attitudes towards diagnosis and management of idiopathic pulmonary fibrosis (IPF) patients in Latin America.

**Methods:**

A Cross-sectional survey was developed and up to 455 physicians were enrolled. We used a rigorous method of validation using the translated version of the AIR Survey.

**Results:**

Mean age was 47.5 years (SD 12.6) with 20.4 years (SD 12.3) of practice. In around 30% of physicians were reported access to radiologist, pathologist and multidisciplinary team. Despite almost all physicians reported that (ATS/ERS/JRS/ALAT) guidelines are useful, half of them prescribed corticoids for treatment of disease. Most respondents (69.9%) reported cough as the presenting symptom. Around 80% considered IPF to be an important clinical disorder, and felt that identifying patients at risk for IPF was important or extremely important. However, only 59.7% felt confident in managing patients with IPF, and similar numbers (60.8%) felt confident about their knowledge. Pulmonologist have more confidence and management of IPF that no pulmonologist.

**Conclusion:**

The results of this survey of Latin American physicians could help to fill gaps regarding awareness, management and treatment of IPF and improve earlier diagnosis of IPF.

**Electronic supplementary material:**

The online version of this article (10.1186/s12890-017-0569-1) contains supplementary material, which is available to authorized users.

## Background

Idiopathic Pulmonary Fibrosis (IPF) is characterised by chronic, progressive and irreversible decline in pulmonary function. Clinical symptoms and natural history are unpredictable and vary in severity [1]. Because of the complex nature and variable course of IPF, diagnosis and treatment are difficult. New data have been published on the safety and efficacy of treatments proposed to slow the progression of the disease, to relieve symptoms, and to improve quality of life [2, 3].

Using narrow case definitions, the annual incidence of IPF in the US is estimated to be 6.8–8.8 per 100,000. IPF incidence increases with age and is higher among males. In recent years the incidence appears to be increasing [4].

A retrospective longitudinal study suggested that median survival of IPF patients is 3–5 years following diagnosis [5].

Confirmation of the diagnosis requires exclusion of other causes of interstitial lung disease (ILD), along with the presence of the characteristic usual interstitial pneumonia (UIP) indicated by honeycombing on high resolution computed tomography (HRCT). In those without definitive radiological UIP, a surgical lung biopsy revealing characteristic findings of UIP is necessary for conclusive diagnosis [6, 7].

The 2011 American Thoracic Society/ Japanese Respiratory Society/ Latin American Thoracic Society (ATS/ERS/JRS/ALAT) guidelines call for a multidisciplinary team (MDT) approach to IPF, in which information from several sources is combined and evaluated [1]. MDT discussion is now considered the gold standard for distinguishing IPF from other ILDs disease [6]. In practice, MDT discussion may not always be possible [8]. Consequently, there is a degree of variability in clinical practice among physicians, both in terms of diagnosis and treatment of IPF [9]**.**

A European survey of IPF management suggested that physicians may benefit from periodic education and update regarding IPF management [10]. In some countries there is a lack of uniformity regarding the diagnosis and management of IPF [11]. On the other hand more of a half trainees physician felt that their ILD training was inadequate [12].

In Latin America, where information is scarce, there is a clear need to standardise the approach to diagnosis and treatment of IPF patients.

The aim of our study was to assess current practice patterns and attitudes towards diagnosis and management of IPF patients in Latin America, to compare specifically pulmonologists and non-pulmonologists related to IPF management.

## Methods

### Study design

We conducted an anonymous cross-sectional survey, in which physicians evaluated themselves regarding idiopathic pulmonary fibrosis. The original questionnaire (AIR Survey, written in English) consisted of 28 questions relating to the clinical management of patients with IPF [8]. We included questions designed to assess physician attitude toward the disease. We used a rigorous method of validation using the translated version of the AIR Survey which we briefly described in the following paragraph.

Two of the investigators translated the AIR survey to Spanish. After that, the Spanish-language version was translated to English by a third investigator who did not know the original version of the AIR Survey. The back-translated English-language version of the new Spanish-language questionnaire was compared with the original English-language version. Each item on the back-translated English-language version was ranked by 30 individuals who were bilingual and independent of the study team for comparability and similarity of interpretability with the same item on the original English-language version. Any translated item with a mean score > 3 (seven was the worst agreement and one was the best agreement) was formally reviewed and corrected. The revised item was then translated back to English and compared again with the original English-language version of that item. This process continued until the mean scores for each item indicated a valid version (63 on each of the comparability and interpretability rankings, and preferably <2.5 on the interpretability rankings) [13].

### Recruitment

The target population included physicians from Ecuador and elsewhere in Latin America who attended the following meetings between November 2015 and October 2016: the VII-VIIIth International Meeting of Allergy and Respiratory Medicine, Guayaquil, Ecuador; and the XIIth Latin-American Expert Forum Santiago, Chile. The inclusion criterion was having received a diploma as a medical doctor. Potential candidates were asked if they often managed patients with IPF. If they answered in the affirmative, they were eligible for the study, and a self-administered survey was delivered. This study was performed in accordance with the Declaration of Helsinki. This human study was approved by Comité de Ética en Investigación en Seres Humanos - Clínica Kennedy. All adult participants provided verbal consent to participate in this study and this was approved by our IRB and was because survey was anonymous and no interventions were conducted. The verbal consent process was recorded/documented by the moment in which physician completed the questionnaire.

### Assessment

The first 28 questions of the AIR survey assessed the current diagnostic and management of IPF, including pharmacological therapy by physicians experienced in IPF and management of comorbidities. Furthermore, we included two “attitude questions” regarding the importance of IPF as a clinical disorder and IPF diagnosis, and three attitude questions regarding the physician’s confidence in diagnosing, managing and knowledge about IPF. These “attitudes and confidence questions” were scored on a five-point Likert scale.

### Statistical analysis

We used mean and standard deviation (SD) for age, years of practice, and for number of patients with IPF attended according to their age and forced vital capacity (FVC). Percentages (%) were calculated for gender, nationality, location of medical’s office, pulmonologist, access to pathologist, radiologist, multidisciplinary team, perception of IPF guidelines, risk factors and onset of disease, treatment, monitoring progression of IPF and managing comorbidities, and attitudes towards disease. Attitudes towards disease were summarized as very important (very important and extremely important) and very confident (agree and strongly agree). Mann-Whitney U tests and chi square were used for comparisons among pulmonologists and non-pulmonologists and number of patients with IPF attended according to their age and FVC. Crude logistic analysis were performed among pulmonologists (non-pulmonologist as reference category) and access to pathologist, radiologist, multidisciplinary team, perception of IPF guidelines, risk factors and onset of disease, treatment, monitoring progression of IPF and managing comorbidities, and attitudes towards disease. Statistical tests were performed using SPSS version 13 (SPSS, Inc., Chicago, IL, 2000). A *p*-value <0.05 was significant.

## Results

Up to 455 physicians were enrolled. The majority were male (55.4%). In total, 23.7% reported being a pulmonologist. Mean age was 47.5 years (SD 12.6) with 20.4 years (SD 12.3) of practice.

Most physicians (74.7%, *n* = 340) reported having cared for an IPF patient in previous year. Among these physicians, 38.6% were female, attending in private settings (48.4%), and one third (29.7%) were pulmonologists. Most of participants were from Ecuador (67.9%), followed by Chile (9.7%) and Peru (8.8%) (Additional file 1: Table S1).

On average, 13.8 (SD 21.4) IPF patients were cared for in the previous year. Physicians reported the highest average number of IPF patients in the age group 66–80 years old (3.6 patients, SD 12.1), with a forced vital capacity (FVC) ranging from 50%–70% (3.3 patients, SD 9.8) (Table 1). Pulmonologist reported the highest number of IPF patients with a FVC ≥50% (*p* < 0.001).

**Table 1:** Comparisons among pulmonologist and non-pulmonologist physicians according to FVC value of IPF patients attended by them

Amount of patients	Pulmonologists						
	Yes *n* = 91 (29.7%)		No *n* = 215 (70.3%)		Total		MW-U*
	Mean	SD	Mean	SD	Mean	SD	*p* value
FVC < 50%	2.75	7.36	1.54	5.10	1.90	5.88	0.214
FVC 50%–70%	7.13	15.40	1.74	5.22	3.35	9.76	0.000
FVC 71%–80%	2.99	8.44	1.10	5.44	1.66	6.52	0.000
FVC > 80%	1.47	5.89	.63	4.67	.88	5.07	0.001

*MW-U: Mann–Whitney U test

### Access to pathologists, radiologists and multidisciplinary teams

Approximately one third of survey respondents reported access to pathologists (27.8%), radiologists (39.4%), and multidisciplinary teams (26.9%). Pulmonologists reported greater access to radiologists (OR 1.59, CI 95% 0.91–2.78) and pathologists (OR 1.80, CI 95% 1.09–2.96). Access to multidisciplinary teams was more common in pulmonologists (41.1% vs 20.8%) compared with non-pulmonologists (p < 0.001).

### Perception regarding IPF guidelines, risk factors and disease onset

ATS/ERS 2011 guidelines were considered useful by almost all respondents (93.7%). No differences were found among among physician specialties (OR 0.44, CI 95% 0.12–1.56). A total of 18.9% of respondents reported that 0–10% of their IPF patients smoke. Pulmonologists reported higher rates of attending patients who smoking (21.3% vs 17.8%, *p* < 0.05). Up to 49.5% were aware that average time between symptom onset and diagnosis was ≤6 months, with no differences among specialties.

Almost one third (39.3%) of respondents reported that their patients had visited one or two physicians prior to diagnosis, with no differences among specialties.

Most respondents (69.9%) reported cough as the presenting symptom. This result held with pulmonologists and non-pulmonologists (OR 1.29, CI 95% 0.75–2.23), and was similar among countries. In addition, dyspnoea was frequently reported (54.6%), more often by pulmonologists (OR 5.22, CI 95% 2.92–9.34)). “Velcro” breath sounds were considered to be the first sign of IPF in 27.9% of respondents, more frequently in pulmonologists (OR 7.93, CI 95% 4.53–13.90).

Up to 92.8% of respondents reported obtaining family history. In total, 97.9% inquired about occupation, while 18.4% pursued genetic causes. Up to 22.3% of respondents reported managing mild and moderate IPF patients themselves, without collaboration. This finding was more frequent among pulmonologists (35.0% vs 17.2%) (*p* < 0.001).

Regarding diagnosis and treatment of patients with mild to moderate IPF, greater than 80% of physicians reported that it was somewhat important (> 3 points) to make and confirm the diagnosis, to manage comorbidities, to develop a monitoring plan, and to monitor efficacy and tolerability of medication. Differences were not found among physician specialties.

### Treatment of IPF

Supplemental oxygen was prescribed most often (71.2%), followed by corticosteroids alone (48.0%), N-acetylcysteine (NAC) alone (29.1%), and pirfenidone (17.0%). Higher chances of supplemental oxygen prescription (OR 2.89; CI 95% 1.53–5.45) and pirfenidone (OR 10.29, CI 95% 5.20–20.39) were observed in pulmonologists (Table 2). Participation in studies with pirfenidone were reported by 10.5% of respondents, with higher chances among pulmonologists (OR 6.54, CI 95% 2.95–14.48) .

**Table 2:** Comparisons among pulmonologist and non-pulmonologist physicians, according to progressionassessment of disease

Treatment	Pulmonologist							
	Yes *N* = 91 (29.7%)		No *N* = 215 (70.3%)			Total		
	n	%	n	%	OR (CI 95%)	n	%	*p* value*
Supplemental oxygen	77	84.6	141	65.6	2.89 (1.53–5.45)	218	71.2	0.001
Corticosteroids (CS)	39	42.9	108	50.2	0.74 (0.45–1.22)	147	48.0	0.238
Azatioprine	5	5.5	13	6.0	0.90 (0.31–2.61)	18	5.9	0.851
CS + immunosupressors (IS)	6	6.6	26	12.1	0.51 (0.20–1.29)	32	10.5	0.151
N-acetylcisteine (NAC)	30	33.0	59	27.4	1.30 (0.77–2.21)	89	29.1	0.331
Corticosteroids + NAC + IS	14	15.4	45	20.9	0.69 (0.36–1.33)	59	19.3	0.261
Corticosteroids + NAC	17	18.7	46	21.4	0.84 (0.45–1.57)	63	20.6	0.591
Colchicine	3	3.3	11	5.1	0.63 (0.17–2.32)	14	4.6	0.486
Ciclosporine	0	0.0	2	0.9	NS	2	0.7	0.356
Anti-blotting	1	1.1	15	7.0	0.15 (0.02–1.14)	16	5.2	0.035
Pirfenidona	38	41.8	14	6.5	10.29 (5.20–20.39)	52	17.0	0.000
Pirferidona + NAC	18	19.8	7	3.3	7.33 (2.94–18.25)	25	8.2	0.000

*Chi square

### Monitoring progression of disease

The most common metrics for assessing progression of disease were dyspnoea (63.7%), followed by FVC (62.1%). Diffusing capacity (DLCO) was used less commonly (24.8%). Similarly, only 28.4% of respondents reported using the 6-min-walk test (6MWT). Pulmonologists reported higher chances of employing for most of these tests (*p* < 0.001) (Table 3).

**Table 3:** Comparisons among pulmonologist and non-pulmonologist physicians, according to progression assessment of disease.

Progression assesment	Pulmonologist							
	Yes n = 91 (29.7%)		No n = 215 (70.3%)			Total		
	n	%	n	%	OR (CI 95%)	n	%	*p* value*
Dyspnea Scale	60	65.9	135	62.8	1.15 (0.69–1.92)	195	63.7	0.601
FVC lowering	75	82.4	115	53.5	4.08 (2.23–7.45)	190	62.1	0.000
DLCO lowering	50	54.9	26	12.1	8.87 (4.95–15.86)	76	24.8	0.000
CT	51	56.0	73	34.0	2.48 (1.50–4.09)	124	40.5	0.000
Exacerbation	28	30.8	65	30.2	1.03 (0.60–1.75)	93	30.4	0.926
6MWT	49	53.8	38	17.7	5.43 (3.16–9.33)	87	28.4	0.000
patient’s feedback	22	24.2	19	8.8	3.29 (1.68–6.44)	41	13.4	0.000

*Chi square test

### Managing comorbidities

In total, 64.1% of respondents reported always investigating for gastroesophageal reflux disease (GERD) (69.7% in pulmonologists vs 61.7% in non-pulmonologists, *p* < 0.05). GERD was considered very important to manage by 48.5% of respondents, but was treated systematically by only 32.3% (37.8% by pulmonologists vs 29.8% by non-pulmonologists). Non-pulmonologists considered managing GERD as very important (55.6%) more frequently than pulmonologists (45.3%).

Diet and lifestyle modifications were commonly recommended (75.7%), followed by proton pump inhibitors (PPI) (69.3%) for treatment of GERD. PPI was more commonly prescribed by pulmonologists (OR 2.67, CI 95% 1.45–4.90)). H2 blockers were prescribed by one quarter of respondents (23.9%). This non-recommended treatment was recommended more commonly by non-pulmonologists (OR 0.53, CI 95% 0.28–1.00)).

The most frequent assessed comorbidity was cardiovascular disease (9.1%), followed by GERD (8.6%), and lung cancer (7.4%). Lung cancer was assessed more frequently among pulmonologists (10.0% vs 6.2%, *p* < 0.05).

Pulmonary Hypertension (PH) was treated in 55.0% of patients. Sildenafil was used by half of respondents (52.5%), more frequently by pulmonologists (OR 2.01, CI 95% 1.22–3.34)).

### Attitudes about IPF

Among respondents that saw at least one IPF patient per year, 77.2% considered IPF to be an important clinical disorder, and 83.5% felt that identifying patients at risk for IPF was important or extremely important. Of these, 65.9% felt confident in identifying patients at risk for IPF. However, only 59.7% felt confident in managing patients with IPF, and similar numbers (60.8%) felt confident about their knowledge (regarding diagnosis and management) of IPF. Pulmonologists reported highest rates of importance and confidence (*p* < 0.001) (Table 4).

**Table 4:** Comparisons among pulmonologist (n = 91, 29.7%) and non-pulmonologist physicians (n = 215; 70.3%) according to attitudes toward disease

Attitudes	Pulmonologist						
	Yes N = 91 (29.7%)		No N = 215 (70.3%)		Total		
	n	%	no	%	n	%	*p* value*
As a clinical disorder, IPF is important	76	86.4	148	73.3	224	77.2	0.014
IPF diagnosis is important	82	93.2	161	79.3	243	83.5	0.003
Confidence about diagnosing IPF	73	83.0	118	58.4	191	65.9	0.000
Confidence about maging IPF	68	77.3	105	52.0	173	59.7	0.000
Confidence about knowledge of IPF	69	78.4	106	53.0	175	60.8	0.000

*Chi square test

## Discussion

In our study, approximately one quarter of Latin American respondents reported access to a pathologist or a multidisciplinary team in their professional network, as compared with European participants (74% and 56%, respectively). Meanwhile, European physicians reported greater access to a radiologist (85%), in our continent this access is only available in one third of physicians [8]. In contrast, pulmonologist have more access to pathologist, radiologist and multidisciplinary teams. Approximately 20% of respondents determine IPF management on their own, compared with 7% reported in the European survey [8].

Earlier diagnosis of IPF has become more relevant since controlled clinical trials have demonstrated a reduction in the rate of decline of FVC [14, 15]. Almost half of respondents reported that the average time between symptoms and diagnosis was ≤6 months, this result differs from European surveys in which the range was 9–12 months. It is possible that our Latin-American IPF patients were actually diagnosed earlier. Alternatively, our result may reflect projection bias.

Our respondents reported that the most common presenting symptom was cough (69.9%). This result was similar across specialties. This result differs from European reports [8], because physician reported that dyspnoea was the most common symptom (99%). However, pulmonologist reported more dyspnoea and that velcro breath sounds were the first sign of IPF. All this could indicate that pulmonologists have more clinical suspicion of IPF. It is possible that our IPFß patients present with a different IPF phenotype.

Our respondents reported that 22% of their patients smoked, a substantially smaller percentage than reported in the European survey (60%) [8]. This finding suggests that there may be other risk factors associated with IPF in our countries. Due to pulmonologists reported higher rates of smoking, it could be possible that non-pulmonologists attend patients with other causes of IPF. Despite of our patients smoke less, they reported cough as the primary onset symptom, and in general present with more aggressive symptoms. Few physicians pursued genetic causes, probably owing to the difficulty of performing this kind of test in our countries.

Because IPF is a disease with a variable clinical course, it is important to identify factors that may help predict prognosis. Published guidelines recommended such tests as FVC, DLCO and 6MWT [16, 17]. The high variability associated with IPF makes predicting prognosis difficult, and this in turn complicates treatment planning. Less than one quarter of respondents employ DLCO and 6MWT, both of which could aid assessment and treatment planning. Is known that physician have low confidence interpreting result investigations in IPF [12]. In our study, the pulmonologists reported higher chances of usage for most of these tests.

Only 10% reported assessing GERD in IPF patients. Half of respondents treated PH patients with sildenafil, in contrast to the AIR survey, in which only a small proportion of respondents (4%) would regularly treat PH. [8].

We predicted that IPF recommendations would be followed by our respondent group because almost half of participants reported they felt IPF guidelines were useful. This was not necessarily the case. First, corticosteroids were frequently prescribed, in combination with immunosuppressive therapy or NAC, contrary to guidelines. A similar discrepancy was found in an Australasian study [11]. However, pulmonologist have higher rates of supplemental oxygen prescription and use of pirfenidone.

Approximately one third of respondents prescribed no specific treatment for IPF, similar to the result found in European surveys [3, 9]. Pirfenidone, a recommended medication according to guidelines, was variably prescribed, possibly because of limited availability in some countries.

There are some disagreements between IPF guideline recommendations and confidence in managing patients with IPF. These discrepancies may reflect a degree of self-protection among respondents.

Our results are comparable to the AIR study because respondents manage IPF patients with similar levels of severity [11]. Establishment of earlier diagnosis in both studies suggests that we may be optimistic regarding the future of IPF. Pulmonologists reported better confidence and manage of IPF.

We suggest that optimal physician management of IPF helps to identify factors that substantially affect patient outcomes. Updated IPF guidelines in local languages could lead to improved collaboration among primary care physicians, pulmonologists, specialised centres, pharmacists and other stakeholders involved in IPF management.

Our study has several limitations. First, this was a cross-sectional survey of physicians attending conferences. Therefore, we cannot infer causation from any of the associations we observed. We cannot generalise our results to all Spanish-speaking physicians because medical education regarding IPF might differ in other Spanish-speaking countries in important ways. Furthermore we have few physician from other Latin-American countries and we cannot generalize our result from all Latinamerica. Physicians who travel to medical meetings are likely to attend educational programs routinely, and therefore these physicians might have access to more updated medical knowledge than the larger group of general practice physicians practicing in the community who do not attend continuing medical education meetings. Thus, management of IPF in the larger population of general practice physicians and pulmonologists is likely to be less optimal than was reported in our survey. Furthermore, survey was self-administered with subjective answers, that could reflect some protection bias. Finally, our study didn’t include radiologists and pathologist, who are members of MDT in diagnosis of IPF.

The results of this survey of Latin American physicians could help to fill gaps regarding awareness, management and treatment of IPF especially in GP. Future studies are needed to validate this survey in Latin America and other Spanish-speaking physician populations. Also, IPF guidelines in clinical practice in Latin America could be implemented according to clinical scenario in our region. Incorporating IPF-focused educational interventions during medical school and residency training programs could help to improve management of the disease. Latin American pulmonology societies could offer more IPF sessions at continuing medical education conferences. They could also create IPF education programs for general physicians such as those that currently exist for asthma and chronic obstructive pulmonary disease.

## Conclusions

Despite high rates of awareness of international IPF guidelines, most IPF recommendations are not followed. To achieve better outcomes for our patients, we recommend improving IPF education in Latin America. Better knowledge of the disease and improved skills in generating a differential diagnosis may contribute to earlier diagnosis and treatment of IPF.

## Additional file

Additional file 1: **Table S1**. Country. (DOCX 12 kb)

## Abbreviations

6MWT: Six-minute walking test
AIR: Advanced IPF Research
ATS/ERS/JRS/ALAT: American Thoracic Society/European Respiratory Society/Japanese Respiratory Society/Asociación Latinoamericana del Tórax
DLCO: Diffusing capacity of Co
FVC: Forced Vital Capacity
GERD: Gastro-Esophageal Reflux Disease
HRCT: High Resolution Computed Tomography
IPF: Idiopathic Pulmonary Fibrosis
PH: Pulmonary Hipertension
SD: Standard Deviation
UIP: Usual Interstitial Pneumonia
