# Supplementary material for: Management and attitudes about IPF (Idiopathic Pulmonary Fibrosis) among physicians from Latin America
Source: BMC Pulm Med. 2018 Jan 10;18:5. doi: 10.1186/s12890-017-0569-1 (PMC5763612; doi:10.1186/s12890-017-0569-1)
Supplement: Additional file 1: — Table S1. Country. (DOCX 12 kb) [file 12890_2017_569_MOESM1_ESM.docx]

Additional file 1

Table S1.

| Country | n | % |
| --- | --- | --- |
| Missing | 26 | 7.6 |
| Argentina | 6 | 1.8 |
| Chile | 33 | 9.7 |
| Colombia | 10 | 2.9 |
| Ecuador | 231 | 67.9 |
| Paraguay | 2 | 0.6 |
| Peru | 30 | 8.8 |
| Uruguay | 2 | 0.6 |
| Total | 340 | 100.0 |
